# Beneficial Effects of New Zealand Blackcurrant Extract on Maximal Sprint Speed during the Loughborough Intermittent Shuttle Test

**DOI:** 10.3390/sports4030042

**Published:** 2016-08-05

**Authors:** Mark ET Willems, Luke Cousins, David Williams, Sam D. Blacker

**Affiliations:** Department of Sport and Exercise Sciences, University of Chichester, College Lane, Chichester, PO19 6PE West Sussex, UK; luke_cousins91@hotmail.com (L.C.); david.williams13@outlook.com (D.W.); s.blacker@chi.ac.uk (S.D.B.)

**Keywords:** sports nutrition, sprinting, football, anthocyanin, polyphenols, fatigue, running performance

## Abstract

New Zealand blackcurrant (NZBC) extract has been shown to enhance high-intensity intermittent treadmill running. We examined the effects of NZBC extract during the Loughborough Intermittent Shuttle Test (LIST) which involves 5 × 15 min blocks with intermittent 15-m maximal sprints, interspersed by moderate and high-intensity running to simulate team sport activity, and a subsequent run to exhaustion. Thirteen males (age: 22 ± 1 year, $\dot{V}O_{2\text{max}}$: 50 ± 5 mL·kg^−1^·min^−1^) participated in three indoor sessions (T: 24 ± 3 °C, humidity: 52% ± 9%). In the first session, a multistage fitness test was completed to determine peak running speed and estimate $\dot{V}O_{2\text{max}}$. Participants consumed NZBC extract in capsules (300 mg·day^−1^ CurraNZ™) or placebo (PL) (300 mg·day^−1^ microcrystalline cellulose M102) for seven days in a double-blind, randomized, cross-over design (wash-out at least seven days). NZBC extract did not affect average 15-m sprint times in each block. NZBC reduced slowing of the fastest sprint between block 1 and 5 (PL: 0.12 ± 0.07 s; NZBC: 0.06 ± 0.12 s; *p* < 0.05). NZBC extract had no effect on heart rate, vertical jump power, lactate and time to exhaustion (PL: 13.44 ± 8.09 min, NZBC: 15.78 ± 9.40 min, *p* > 0.05). However, eight participants had higher running times to exhaustion when consuming NZBC extract. New Zealand blackcurrant extract may enhance performance in team sports with repeated maximal sprints.

## 1. Introduction

Many field sports (e.g., football, rugby and hockey) require players to perform intermittent activity patterns with alternating periods of near maximal and maximal running sprints and submaximal exercise recovery (e.g., walking and jogging) [1]. For example, in the analysis of televised matches of professional football games, it was observed that midfield players performed sprints for 6.4% of purposeful movement [2]. In addition, Aslan et al. [3] used a match analysis system over 2 × 45 min of friendly football matches between young players (with 3-min breaks every 15 min to take lactate) and observed that 3.3% of the total distance in both halves was covered by high-intensity sprints. In professional football, a disproportionate number of goals are scored in the last 15 min of match play [4], when players are likely experiencing physical fatigue. Therefore, an ability to maintain the performance of repeated maximal sprints in field sports, especially late on in the game, may affect game play and outcome.

The Loughborough Intermittent Shuttle Test (LIST) was designed to replicate the activity patterns of football [5] with the performance and physiological responses mimicking real game demands such as distance covered [6,7], number of sprints [8], heart rate [9,10] and blood lactate concentrations [6,9]. In general, the mechanisms of fatigue by exercise depend on the intensity and duration when the exercise is sustained, with an additional influence of recovery time with intermittent exercise. During the LIST, fatigue may be caused by phosphocreatine hydrolysis with insufficient phosphocreatine recovery due to the availability of oxygen [11] and the ability to buffer metabolites (e.g., hydrogen) [12,13,14], accumulation of metabolites [1] with a potential role of oxidative stress by the production of free radicals [15].

Logistical issues and unpredictable requirements of real play field sports complicate the study of the potential effect of ergogenic aids on the physical, physiological, and metabolic demands during a game. Numerous studies have therefore examined the ergogenic effects of a nutritional intervention on the responses during the LIST [16,17,18,19]. Four weeks of β-alanine supplementation did not improve sprint performance during the LIST [19], although β-alanine supplementation is recognised to increase muscle carnosine concentration responsible for increased hydrogen buffering [20]. Sprint performance improved with a carbohydrate-electrolyte supplementation during the LIST, potentially explained by a greater concentration of blood glucose than under the placebo condition [16]. The effectiveness of a polyphenol supplementation on responses during the LIST has not been examined.

Blackcurrant contains multiple anthocyanins that display anti-inflammatory [21] and anti-oxidant activity [22] with the potential to counteract the negative effects of high-intensity intermittent exercise. Blackcurrant intake may have attenuated the exercise-induced reactive oxygen species (ROS) generating capability during 30-min rowing [23], reducing the potential negative effects of ROS on the skeletal muscle sodium-potassium pump, and diminishing the onset of fatigue [24]. In addition, anthocyanins alter endothelial function by modulation of endothelial nitric oxide synthase (eNOS), the enzyme involved in the metabolism of the vasodilator nitric oxide (NO) [25]. Blackcurrant intake increased peripheral blood flow in the forearm by up to 22% during typing [26], most likely through anthocyanin induced vasorelaxation and vasodilation as observed in the thoracic aortic rings of male Wistar rats [27]. However, caution is required when comparing in vitro findings to an in vivo context in humans. Increased blood flow during high-intensity intermittent exercise, however, may allow for greater lactate and hydrogen removal, accelerating phosphocreatine resynthesis by improved oxygen availability [11] and, thus, delaying fatigue [28]. Perkins et al. [29] observed in a treadmill test that intake of New Zealand blackcurrant (NZBC) extract increased the ability to complete an increased number of intermittent high-intensity runs resulting in a 10.8% increase in total running distance. In that study, the high-intensity runs were never at maximal running speed. It is not known whether there is an ergogenic effect of New Zealand blackcurrant supplementation on maximal sprint speeds during the LIST.

Therefore, the aim of the present study was to examine the effect of New Zealand blackcurrant extract on sprint performance, heart rate and lactate responses during the LIST. In addition, we examined vertical jump performance as an indicator of the presence of fatigue during and on completion of the LIST. It was hypothesized that seven days of New Zealand blackcurrant extract consumption would allow better preservation of maximal sprint ability, reduce fatigue, and increase the running time to exhaustion.

## 2. Materials and Methods

### 2.1. Participants

Thirteen healthy males (mean ± SD, age: 22 ± 1 years, body mass: 77.4 ± 8.0 kg, height: 179.3 ± 5.8 cm, $\dot{V}O_{2\text{max}}$: 49.6 ± 5.1 mL·kg^−1^·min^−1^) volunteered to take part in the study. Participants did not receive payment. Informed written consent was obtained after providing verbal and written information on the experimental procedures, supplementation, benefits and potential risks of the study. Participants were recreationally active males with the majority having experience in sports that involve high-intensity intermittent running. Ethical approval for the study was obtained from the University of Chichester ethics committee (code: 1415_40).

### 2.2. Experimental Procedures

The study required participants to attend for three visits. In the first visit, a multistage fitness test (MSFT) was completed to estimate maximum oxygen uptake (i.e., $\dot{V}O_{2\text{max}}$) and establish peak running speed. For the MSFT, participants ran 20-m distances back and forth between two lines, touching the line with a foot each time at the audio signal emitted from a CD player. The time between audio signals decreased each minute. The MSFT ended when the participants could no longer keep up with the required pace. The last completed level was used to estimate $\dot{V}O_{2\text{max}}$ [30]. Peak running speed for each participant was taken as the speed of the last completed level with the average peak running speed 13.2 ± 0.8 km·h^−1^. The peak running speed of each participant was used to stipulate running speeds in the Loughborough Intermittent Shuttle Test (i.e., LIST; see Section 2.2.1. for LIST details), corresponding to speeds at 55% and 95% of peak running speed. After the MSFT, participants were familiarized with two 15 min blocks of part A of the LIST followed by the run to exhaustion, i.e., part B of the LIST [5]. In the second and third visit, the LIST was performed with a double-blind, randomized, cross-over study design. At least a 7-day washout period was prescribed for the 7-day intake of either placebo or NZBC extract (see Section 2.2.2 for details). Before visit two, the dietary intake was recorded for 24 h (Table 1) and replicated for the third visit.

Participants were instructed not to consume alcohol and caffeine or participate in strenuous physical activity for 24 h before the LIST sessions. LIST sessions were completed at the same time of day with water provided ad libitum. Sessions were completed in an indoor sport facility (T: 23.7 ± 2.7 °C, humidity: 52% ± 9%).

#### 2.2.1. Loughborough Intermittent Shuttle Test

Participants completed a 10 min warm up protocol including running at speeds (but not maximal) used in the LIST and self-selected static stretches. Subsequently, participants completed the LIST protocol [5]. The LIST consists of a part A and B, each part completed between two lines that were 20-m apart. Part A requires the participants to complete 5 × 15-min blocks with 3-min recovery between blocks. Each 15-min block consists of successive completion of 3 × 20-m at 1.33 m·s^−1^, 1 × 20-m of maximal sprinting, 4 s recovery, 3 × 20-m running at a speed corresponding to 55% peak running speed and 3 × 20-m running at 95% peak running speed (determined by the MSFT). Depending on the individualised running speeds, each 15-min block involved 9 or 10 maximal sprints. The times required for the running speeds during the LIST were set by an audio signal recording for each participant. Maximal sprint times were recorded in one direction over the first 15-m [5] using Fusion Smartspeed lightgates (HaB International, Warwickshire, UK). Heart rate was recorded every 5 s throughout the experimental sessions (Polar RS800 heart rate monitors, Polar Electro UK Ltd., Warwick, UK).

During the 3-min recovery after each 15-min block, a fingertip blood sample was collected and analyzed for blood lactate with a portable analyzer (Lactate Pro, Birmingham, UK). In addition, participants performed three maximal vertical jumps using an in-house designed jump mat to calculate vertical jump power from the two highest jumps and allow a measure of fatigue. For the vertical jumps, participants kept hands on the hips with knees bent around 90 degrees before jumping. Jumping was initiated about 10 to 15 s after completion of a block in part A and on completion of part B. The last block of 15 min of part A (i.e., block 5) of the LIST was also followed by a 3-min recovery with blood sampling and jumping before initiating part B. Part B of the LIST consisted of running 20 m at alternating speeds of 55% and 95% of peak running speed. Part B was stopped when the participant was unable to reach the 20-m line on two consecutive occasions at a speed of 95% of peak running speed.

#### 2.2.2. Supplementation

Participants took one 300 mg capsule a day for 7 days of New Zealand blackcurrant extract (containing 105 mg of anthocyanin, i.e., 35%–50% of delphinidin-3-rutinoside, 5%–20% of delphinidin-3-glucoside, 30%–40% of cyanidin-3-rutinoside, and 3%–10% of cyanidin-3-glucoside) (CurraNZ™, Health Currancy Ltd., Surrey, UK), or placebo (PL) (i.e., 300 mg microcrystalline cellulose M102). Participants were instructed to consume the last capsule 3 h prior to testing, arrive hydrated by drinking water only and consume a light breakfast/lunch ≥2 h prior to LIST testing. The optimal dosing strategy for New Zealand blackcurrant extract is not known, but a similar dosing strategy was used in studies that observed performance improvements for a cycling time trial [31] and high-intensity treadmill running [29].

### 2.3. Statistical Analyses

Statistical analyses were completed using statistical package SPSS v20.0 (SPSS Inc., Chicago, IL, USA). Data normality was assumed following Kolomogrov-Smirnov tests and analysis of skewness and/or kurtosis. Differences in sprint times, vertical jump power, blood lactate and heart rate were assessed between conditions (PL vs. NZBC) over the time points using a two-way repeated measure ANOVA, with post-hoc paired t-tests for significant condition effects and Bonferroni’s multiple comparisons test for significant time effects. Paired samples t-tests were conducted to compare for between placebo and NZBC conditions slowing of the fastest sprint time of block 1 in each of the consecutive blocks 2–5, running times of part B of the LIST, and 24 h dietary intake. Statistical significance was accepted at *p* < 0.05. Interpretation of 0.05 > *p* ≤ 0.1 was according to guidelines by Curran-Everett and Benos [32]. All data are reported as means ± SEM.

## 3. Results

### 3.1. Loughborough Intermittent Shuttle Test—PART A

#### 3.1.1. Fifteen-Meter Sprint Times

**Figure 1:**
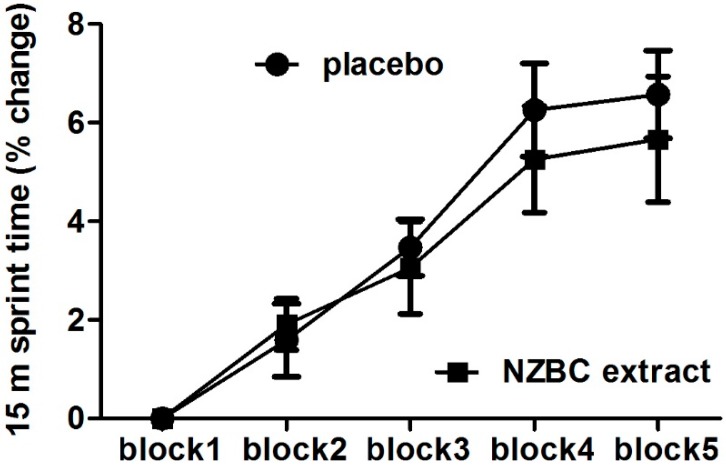
Change in average maximal 15-m sprint times during blocks 2–5 (part A) of the LIST. Sprint times in blocks 2–5 are expressed as a percentage change of block 1. Data are mean ± SEM.

**Figure 2:**
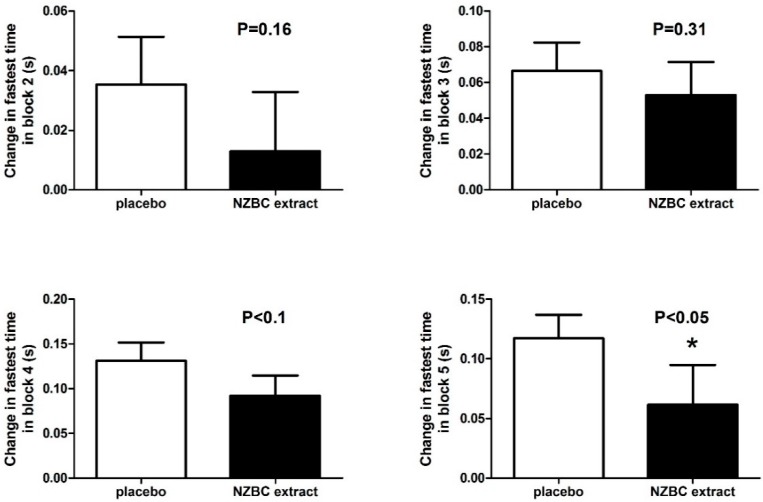
Time of slowing of the fastest sprint time in blocks 2–5 compared to block 1. NZBC, New Zealand blackcurrant. Data are mean ± SEM.

The percentage change in the average 15-m sprint times during the LIST showed a time (*p* < 0.001) but no condition (*p* = 0.63) and interaction effect (*p* = 0.78) (Figure 1). An analysis of the slowing of the fastest 15-m sprint time in block 1 showed for the NZBC condition compared to the placebo condition a trend for less slowing in block 4 (*p* < 0.1) and significant less slowing in block 5 (*p* < 0.05) (Figure 2).

#### 3.1.2. Vertical Jump Power

**Figure 3:**
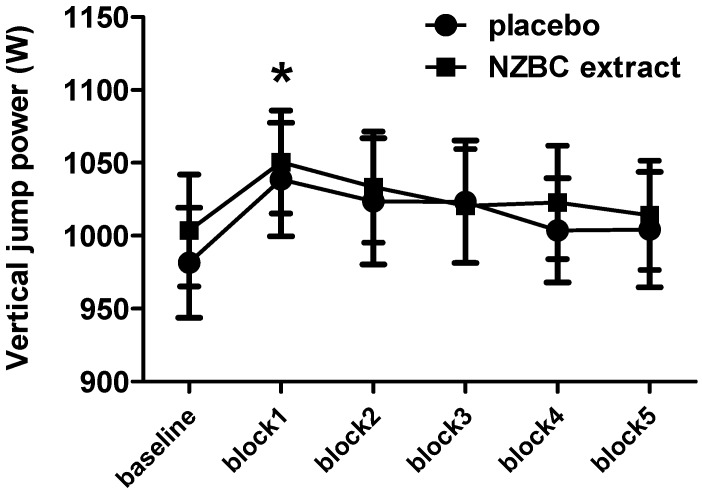
Vertical jump power in the 3-min recovery times between 15-min blocks in part A of the LIST. NZBC, New Zealand blackcurrant. *, different from baseline. Data are mean ± SEM.

Vertical jump power showed a time (*p* < 0.001) but no condition (*p* = 0.83) or interaction effect (*p* = 0.70) (Figure 3). For the time effects in both conditions, vertical jump power was higher in block 1 compared to baseline but there were no differences between vertical jump powers in each block, indicating that in both conditions vertical jump power was maintained during part A of the LIST.

#### 3.1.3. Heart Rate

**Figure 4:**
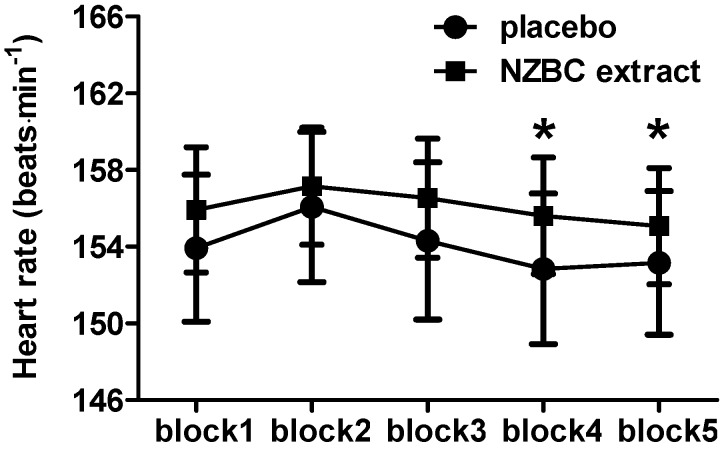
Heart rate during the first five blocks of part A of the LIST. NZBC, New Zealand blackcurrant. *, different from block 2 for placebo. Data are mean ± SEM.

Heart rate data during the LIST showed a time (*p* = 0.02) but no condition (*p* = 0.69) and interaction effect (*p* = 0.90) (Figure 4). Post hoc analysis of the time effects for the placebo condition heart rate to be lower in blocks 4 and 5 compared to heart rate in block 2.

#### 3.1.4. Lactate

**Figure 5:**
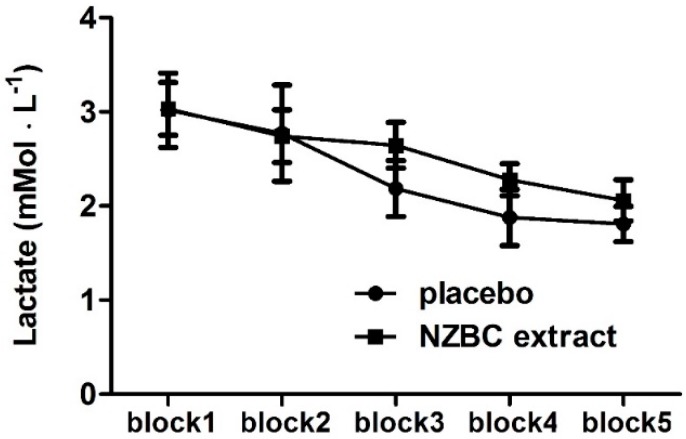
Blood lactate during part A of the LIST. NZBC, New Zealand blackcurrant. Data are mean ± SEM, *n* = 11.

Blood lactate values during part A of the LIST showed a time (*p* < 0.001) but no condition (*p* = 0.44) and interaction effect (*p* = 0.25) (Figure 5). Post hoc analysis of the time effects with Bonferroni multiple comparisons showed for both conditions lower lactate values in blocks 4 and 5 compared to block 1.

### 3.2. The Loughborough Intermittent Shuttle Test—PART B

#### 3.2.1. Time to Exhaustion

**Figure 6:**
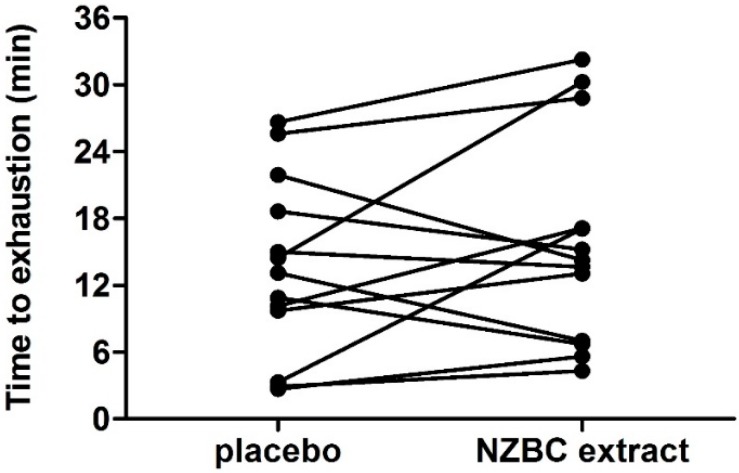
Individual times to exhaustion for part B of the LIST. NZBC, New Zealand blackcurrant.

There was no difference in time to exhaustion (TTE) between conditions during part B of the LIST (placebo: 13.44 ± 8.09 min; NZBC extract: 15.78 ± 9.40 vs., *p* = 0.26) (Figure 6). Eight participants (out of 13) improved the time to exhaustion with NZBC extract.

#### 3.2.2. Vertical Jump Power, Heart Rate and Lactate

In the NZBC condition, heart rate was higher at the end of part B of the LIST (placebo: 171 ± 11 beats·min^−1^, NZBC extract: 176 ± 8 beats·min^−1^, *p* = 0.02). There were no differences in vertical jump power (placebo: 990 ± 151 W, NZBC extract: 992 ± 154 W) and lactate between conditions after part B of the LIST (placebo: 2.99 ± 1.81 mMol·L^−1^, NZBC extract: 2.62 ± 0.86 mMol·L^−1^).

## 4. Discussion

This is the first study that examined the effect of a polyphenol supplement on the performance of an established field test that was designed to simulate the activity pattern of football. The main finding of less slowing of the fastest maximal sprint in the Loughborough Intermittent Shuttle Test with intake of New Zealand blackcurrant extract may indicate that the participants experienced less fatigue. However, we did not observe differences in vertical jump power as our objective indicator of physical fatigue. In the present study, the vertical jump test may not have been sensitive enough to detect physical fatigue from the LIST running requirements by the participants. Some participants seem to respond substantially to the New Zealand blackcurrant intake with an increased ability to extend the run to exhaustion but no group effects were shown. It would have been of interest to know whether such high response would be repeatable. This large variation in the run time to exhaustion, however, does not allow conclusive remarks on this performance aspect of the Loughborough Intermittent Shuttle Test.

Previous studies using the LIST protocol observed participants TTE to be ≤6.5 min [5,18,33]. This is substantially less than in the present study where the mean TTE was 15.8 ± 9.4 min (NZBC, range: 4.3–32.22 min) and 13.4 ± 8.1 min (PL, range: 2.7–26.6 min). In addition, in previous studies, blood lactate responses of 9.3 ± 1.7 mMol·L^−1^ [18] and ~7 mMol·L^−1^ [5] were higher than in the present study (NZBC: 2.6 ± 0.8 mMol·L^−1^, PL: 2.9 ± 1.8 mMol·L^−1^). This observation of longer TTE may be due to a potential low peak running speed reached by the participant during the MSFT in the present study.

The fatigue mechanism during the LIST may involve effects of accumulation of hydrogen and subsequent lowering of muscle pH, due to the repeated bouts of high/maximal intensity performance [34]. Repeated sprint ability has been strongly correlated with the ability to buffer hydrogen in women [12,35]. Notwithstanding buffering of hydrogen, intracellular acidosis reduces the rate of phosphocreatine resynthesis and glycolysis [14]. It also effects muscle contractile properties, reducing muscle excitability by increasing extracellular potassium and intracellular sodium [24], and also increasing intracellular chloride concentration, modulating the voltage-gated chloride channel, further reducing muscle excitability [36]. It is recognized that games players of a high standard have a much better hydrogen buffering capacity than those at a lower standard [13,37]. The participants in the present study were low standard games players and therefore any improvement in hydrogen buffering capacity as a result of NZBC may be evident for the improvement of repeated sprint performance, however, this was not measured. However, anecdotal information collected following completion of the LIST indicated that ten of the participants felt completing the LIST whilst on supplement B (NZBC extract) was much easier than when on the placebo. Price and Moss [38] reported that large accumulations of intracellular hydrogen in high-intensity intermittent exercise to be associated with an increased perception of effort. Future research may want to examine the effects NZBC on hydrogen buffering capacity and intracellular muscle pH.

Another potential fatigue mechanism during the LIST may be linked with the occurrence of oxidative stress [39]. Oxidative stress induced fatigue events include effects on muscle contractile properties, calcium uptake in the sarcoplasmic reticulum, potassium influx into muscle cells, interfering with muscle excitation-contraction coupling and the ability to create action potentials [40]. The antioxidant activity of anthocyanin-rich blackcurrant attenuated ROS generating capability in 30 min of rowing, compared to the placebo, which documented a 1.4-fold increase in ROS generating capability [23]. Such antioxidant activity may have contributed to the increase the TTE in some subjects with NZBC compared to PL conditions.

With NZBC extract intake in the present study, participants were able to better maintain their sprint performance without a significant effect between placebo and NZBC conditions on the absolute sprint times at comparable time-points during the LIST. In a previous study on the effects of NZBC extract on high-intensity running, using a similar dosing strategy, the distance to perform high-intensity runs was increased by 10.8% [29]. However, the exercise modalities in the present study and Perkins et al. [29] were different in intensity and duration of the performance runs in addition to the exercise intensity and duration of the recovery between the performance runs. Recovery periods between repeated sprints influence the reduction in intracellular pH and phosphocreatine resynthesis [12,35] with passive recovery more beneficial during 10 × 30 m sprints [41]. Perkins et al. [29] used 15 s active recovery in a block of 6 high-intensity runs with 60 s passive recovery between the blocks and high-intensity running times to be much longer than in the present study. It is, therefore, likely that higher levels of intracellular disturbances were experienced by the participants in Perkins et al. [29]. In addition, if NZBC would have an effect on blood flow during recovery, it is likely that participants in Perkins et al. [29] may have experienced recovery that benefitted repeated high-intensity running. The recovery times in the present study were much larger with sprint times being drastically shorter; therefore, it would be expected that sprint performance in the LIST was maintained following NZBC extract by reducing fatigue through increased peripheral blood flow, oxygen availability and reduction in oxidative stress. In addition, it is also possible that repeated 15-m sprints with long inter-sprint recovery times were not sufficient to cause considerable intracellular disturbances, but that fatigue occurred due to the duration of the test.

We provided evidence that NZBC extract allowed better maintenance of the fastest sprint time during the Loughborough Intermittent Shuttle Test. In addition, it seemed that some participants were able to increase their time to exhaustion during the exhaustive block of the LIST, on average with those participants running for 15% (i.e., 2 min and 20 s) longer. Participants also had a higher heart rate (*p* = 0.02) during this exhaustive block under the NZBC condition possibly indicating that participants in the NZBC condition were able to tolerate higher heart rate values and able to run for longer with high-intensity before exhaustion. However, aerobic fitness in the present study ($\dot{V}O_{2\text{max}}$: 49.6 ± 5.1 mL·kg^−1^·min^−1^) was lower than observed in elite games players ($\dot{V}O_{2\text{max}}$: 59.4 ± 2.6 and 58.6 ± 2.4 mL·kg^−1^·min^−1^) [19], thus caution is required to generalize our observations for this population.

## 5. Conclusions

It is concluded that the intake of New Zealand blackcurrant extract may be beneficial for performance in team sports that involve maximal sprints with the benefits applicable in the later stages of the game.

## Figures and Tables

**Table 1:** Nutritional intake of participants for placebo and New Zealand blackcurrant (NZBC) extract conditions.

	Calories (kcal)	Protein (g)	Carbohydrates (g)	Fat (g)	Fibre (g)	Sugar (g)
Placebo	2625 ± 642	131 ± 81	287 ± 73	88 ± 29	27 ± 15	82 ± 41
NZBC extract	2573 ± 689	121 ± 67	272 ± 70	85 ± 36	28 ± 10	71 ± 29

No difference between conditions. Values are means ± SD.

## Abbreviations

The following abbreviations are used in this manuscript:

LIST	Loughborough Intermittent Shuttle Test
MSFT	Multistage fitness test
NZBC	New Zealand blackcurrant
PL	Placebo
